# Genetic Diversity, Population Structure, and Botanical Variety of 320 Global Peanut Accessions Revealed Through Tunable Genotyping-by-Sequencing

**DOI:** 10.1038/s41598-018-32800-9

**Published:** 2018-09-28

**Authors:** Zheng Zheng, Ziqi Sun, Yuanjin Fang, Feiyan Qi, Hua Liu, Lijuan Miao, Pei Du, Lei Shi, Wei Gao, Suoyi Han, Wenzhao Dong, Fengshou Tang, Feng Cheng, Haiyan Hu, Bingyan Huang, Xinyou Zhang

**Affiliations:** 1 Industrial Crops Research Institute, Henan Academy of Agricultural Sciences/Key Laboratory of Oil Crops in Huanghuaihai Plains, Ministry of Agriculture/Henan Provincial Key Laboratory for Genetic Improvement of Oil Crops, Zhengzhou, 450002 China; 2 Institute of Vegetables and Flowers, Chinese Academy of Agricultural Science, Beijing, 100081 China

## Abstract

Cultivated peanut (*Arachis hypogaea* L.) were classified into six botanical varieties according to the morphological characteristics. However, their genetic evolutionary relationships at the genome-wide level were still unclear. A total of 320 peanut accessions, including four of the six botanical varieties, and 37,128 high-quality single nucleotide polymorphisms (SNPs) detected by tunable genotyping-by-sequencing (tGBS) were used to reveal the evolutionary relationships among different botanical varieties and verify the phenotypic classification. A phylogenetic tree indicated that the tested accessions were grouped into three clusters. Almost all of the peanut accessions in cluster C1 belong to var. *fastigiata*, and clusters C2 and C3 mainly consisted of accessions from var. *vulgaris* and subsp. *hypogaea*, respectively. The results of a principal component analysis were consistent with relationships revealed in the phylogenetic tree. Population structure analysis showed that var. *fastigiata* and var. *vulgaris* were not separated when K = 2 (subgroup number), whereas they were clearly divided when K = 3. However, var. *hypogaea* and var. *hirsuta* could not be distinguished from each other all the way. The nucleotide diversity (π) value implied that var. *vulgaris* exhibited the highest genetic diversity (0.048), followed by var. *fastigiata* (0.035) and subsp. *hypogaea* (0.012), which is consistent with the result of phylogenetic tree. Moreover, the fixation index (*F*_*ST*_) value confirmed that var. *fastigiata* and var. *vulgaris* were closely related to each other (*F*_*ST*_ = 0.284), while both of them were clearly distinct from var. *hypogaea* (*F*_*ST*_ > 0.4). The present study confirmed the traditional botanical classifications of cultivated peanut at the genome-wide level. Furthermore, the reliable SNPs identified in this study may be a valuable resource for peanut breeders.

## Introduction

Cultivated peanut (*Arachis hypogaea* L.) is one of the most important grain legume crops worldwide, and is a good source of food, vegetable oil, feedstock, and ground cover^1^. Cultivated peanut, with a total genome size of approximately 2.7 Gb, is an allotetraploid (2n = 4X = 40) plant species derived from a single recent hybridization event involving two diploid species, *Arachis duranensis* (A genome) and *Arachis ipaensis* (B genome), followed by a polyploidisation event^2^. On the basis of morphological features, crossing experiments, and seed protein electrophoretic profiles, Krapovickas and Gregory classified cultivated peanut into two subspecies (subsp. *hypogaea* and subsp. *fastigiata*) and six botanical varieties, which include var. *hypogaea*, var. *hirsuta* (within subsp. *hypogaea*), besides var. *fastigiata*, var. *aequatoriana*, var. *peruviana*, and var. *vulgaris* (within subsp. *fastigiata*)^3^. However, these classifications were based on growth habits, morphological features, as well as seed, pod, and inflorescence characteristics^4^. For example, the main distinguishing characteristic between subspecies *hypogaea* and *fastigiata* is the presence of main axis flowers. This trait may be abnormal in some peanut accessions because of genetic contamination (during the outcrossing history of the pedigree). To our knowledge, due to lack of specific description, some varieties have been classified as “irregular type” to distinguish between these accessions (obtained *via* hybridisations between subspecies or crosses between different peanut types) and the unadulterated accessions^5^. The irregular peanut types are probably the result of breeding activities that attempted to increase genetic variation. Crosses involving cultivated peanut are easier than intraspecific hybridisations with wild relatives.

Although broad genetic variation is crucial for cultivar improvement, information regarding genetic diversity is also critical for germplasm utilisation^6^. Several recent studies have applied different marker technologies to analyse the genetic diversity and population structure of specific cultivated peanut populations. For example, 32 highly polymorphic simple sequence repeat (SSR) primer pairs were used to investigate 96 peanut genotypes mainly from the US peanut mini-core collection^4^. Similarly, 146 SSR markers were used to study 196 major peanut cultivars extensively planted in different regions in China^6^, while 111 SSR markers were applied to examine 79 peanut cultivars and breeding lines from China, India, and the US^7^. However, these previous studies included relatively few, primarily SSR markers or an limited number of peanut accessions for evaluations of the genetic diversity and population structure. Moreover, the relationships among 280 genotypes that originated from a reference set of 300 genotypes representing 48 countries were explored using 73 Kompetitive Allele Specific PCR (KASP) genotyping assays^8^. The most recent studies applying next-generation sequencing technology to explore the molecular footprint of agronomic traits related to domestication included an analysis of 158 Chinese peanut accessions based on specific-locus amplified fragment sequencing (SLAF-seq)^9^ and an investigation of the genetic architecture of a reference set of 300 peanut germplasms with the high-density single nucleotide polymorphism (SNP) array ‘Axiom_*Arachis*’ (58 K SNPs)^10^. To the best of our knowledge, there is a lack of molecular genetic evidence of the differences and relationships among cultivated peanut subspecies and botanical varieties based on diverse peanut germplasms and high-density polymorphic loci.

Simple sequence repeat markers, which are associated with high polymorphism information content and represent useful molecular tools for analysing genomes^11^, have been extensively used for assessing the genetic variation of peanut germplasms^4,6,7^. However, these markers have failed to detect a high degree of polymorphism in peanut germplasms because of the extensive repetitive genomic content. Additionally, compared with other marker systems, SSR-based assays are labour-intensive and time-consuming. Thus, a SNP marker assay may be an appropriate alternative because SNPs represent the most abundant DNA sequence variations in the genome. Recently, various high-throughput and relatively inexpensive SNP detection platforms as well as next-generation sequencing techniques have been developed^12–14^. Genotyping-by-sequencing (GBS), which is one of the most important methods for detecting SNPs, is a genome-reduction technique based on the high-throughput next-generation sequencing of genomic subsets targeted by restriction enzymes^15,16^. Tunable GBS (tGBS)^17–20^ is a novel genome-reduction method that involves the ligation of single-stranded oligos to restriction enzyme fragments. Because of the additional (selective) nucleotides at the 3′-end of PCR primers, fewer sites are sequenced in tGBS (i.e., a higher “genome-reduction level”), resulting in an increased read depth per sequenced site, assuming that equal amounts of sequencing data are generated^17^. Compared with the SNPs from conventional low-coverage GBS methods, those generated by tGBS can be called with a higher confidence and do not require imputation, which eliminates or decreases the number of induced errors^17,18^.

The objective of this study was to interpret the evolutionary relationships among different botanical varieties of peanuts and detect numerous potential polymorphic loci. Two varieties in subsp. *fastigiata* (var. *fastigiata* and var. *vulgaris*) were distinguished by analysing 320 peanut germplasms with 37,128 high-quality SNP loci identified by tGBS. Additionally, the botanical varieties of some accessions among the 320 cultivars were redefined according to molecular data and morphological features. This data set will serve as a valuable resource for fundamental research regarding genetic variations in peanuts as well as a source of potential polymorphic loci for the application of marker-assisted selection in peanut breeding. Moreover, we revealed that different botanical varieties can be defined based on molecular information as well as morphological features. Furthermore, the genetic relationships may be useful for identifying and explaining the genetic contaminants in some breeding lines or released varieties, including the imprecisely defined irregular peanut types.

## Results

### Data generation

Strict criteria were used to filter the raw data, and 37,128 high-quality SNPs with less than 30% missing data were analysed regarding their genetic diversity and population structure. Data2Bio conducted tGBS on the 320 samples using ten Illumina HiSeq. 2000 lanes, generated 1.14 × 10^9^ reads. Approximately 1.13 × 10^9^ reads (i.e., 96.6%) remained after eliminating low-quality reads with a PHRED quality score <15 out of 40^21,22^.

The genome of the tetraploid cultivated peanut has not been published. Thus, the genomes of two diploid peanut progenitors, *A*. *duranensis* and *A*. *ipaensis* (Aradu_v1.0.fa and Araip_v1.0.fa)^2^, were artificially combined to form a reference genome for sequence alignments with the GSNAP aligner^23^. Approximately 85.5% of the trimmed reads were aligned to a single genomic location, and were used to detect SNPs. The criteria for identifying homozygous and heterozygous SNPs were as follows: Number of reads ≥ 4; allele frequency ≥ 0.8; exclude the first and last 3 bp of each read; and include polymorphic sites with a PHRED quality score ≥20 (≤1% error rate). Additionally, heterozygous SNPs required an allele frequency ≥0.3. A total of 1,240,787 polymorphic sites, including all sites that differed from the reference sequence in at least one sample, were obtained after aligning all reads to the reference genome.

A subset of 253,595 SNPs was identified, with each SNP required to have less than 80% missing data across 320 samples, and be present in exactly two alleles and in at least two genotypes. The minor allele needed to be present in at least three samples and at least one homozygous sample. An additional requirement was a heterozygosity frequency of 0–0.1. There were 37,128 SNPs that remained when the missing data rate was increased to 30%, while the other filter parameters were unchanged. The histograms presenting the average missing data rate (18.1%) and average number of reads (nine) per SNP site are provided in Supplementary Fig. S1. Because of the scaffold nature of the current reference genome, 33,997 SNPs (i.e., 91.6%) were located on 20 chromosomes. Moreover, the 33,997 SNPs were unevenly distributed across the whole genome (Supplementary Fig. S2) and the number of SNP on each chromosome and SNP density were showed in Supplementary Table S1. There were also more SNPs in the A sub-genome (20,967) than in the B sub-genome (13,030). Furthermore, chromosome A01 harboured the most SNPs (5,119), while chromosome A08, which was the shortest chromosome, contained the fewest (315).

### Genetic distances between peanut accessions

**Figure 1:**
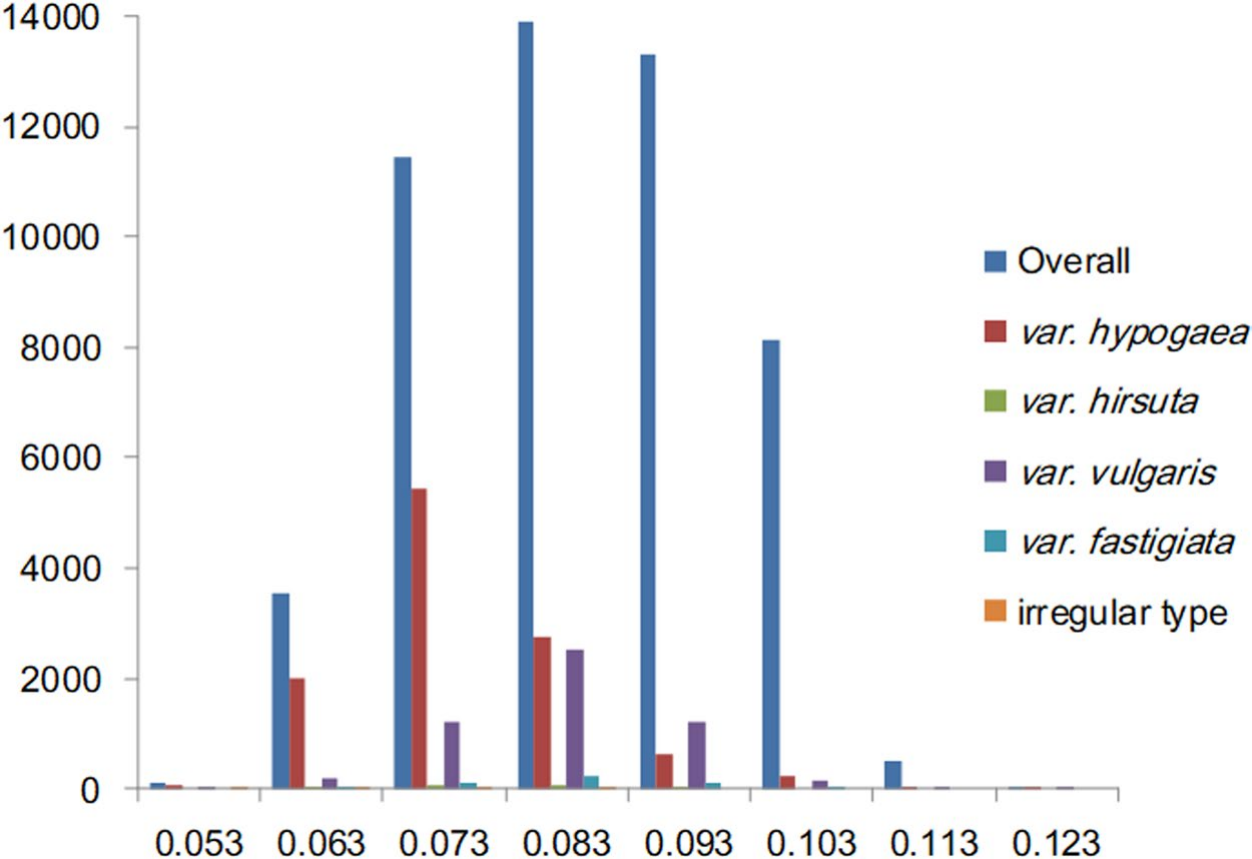
Frequency of genetic distance for different botanical types. The blue, red, green, purple, light blue and orange stand for overall, var. *hypogaea*, var. *hirsuta*, var. *vulgaris*, var. *fastigiata*, and irregular type respectively. For overall, var. *vulgaris* and var. *fastigiata*, most of the genetic distance lie in 0.073~0.083, while for var. *hypogaea*, var. *hirsuta* and irregular type, most of the genetic distance is between 0.063 and 0.073.

The genetic distance matrix between all 320 peanut accessions was calculated used the arithmetic implemented in the software TASSEL5.2.13^24^. Among the 51,040 combinations (Supplementary Table S2), the genetic distance ranged from 0.043 to 0.127, with an average of 0.080 (Table 1). The smallest genetic distance (0.043) was observed between accessions Z142 and Z693, both of which are cultivars from Shandong province, China. An analysis of the pedigrees of these two cultivars revealed that Z693 was the ancestral parent of Z142 (Supplementary Fig. S3). The largest genetic distance (0.127) was detected between accessions Z409 and Z209, which are a var. *hypogaea* released variety from China and a var. *vulgaris* cultivar from South Africa, respectively. For the five tested botanical types, the mean of genetic distance was less than 0.1 and the largest average genetic distance was observed for var. *vulgaris*, followed by var. *fastigiata*, var. *hypogaea*, var. *hirsuta*, and irregular type (Table 1).The genetic distance frequencies for the different botanical types are presented in Fig. 1. The genetic distance between var. *vulgaris* and var. *fastigiata* was between 0.073 and 0.083. In contrast, the genetic distance was between 0.063 and 0.073 for var. *hypogaea*, var. *hirsuta*, and irregular type accessions (Fig. 1).

**Table 1:** Pairwise genetic distance among peanut accessions of different botanical types in the population.

Botanical type	Min	Max	Mean
Overall	0.043	0.127	0.080
var. *hypogaea*	0.044	0.117	0.070
var. *hirsuta*	0.054	0.090	0.069
var. *vulgaris*	0.050	0.117	0.078
var. *fastigiata*	0.054	0.099	0.077
irregular type	0.051	0.078	0.065

### Phylogenetic tree

**Figure 2:**
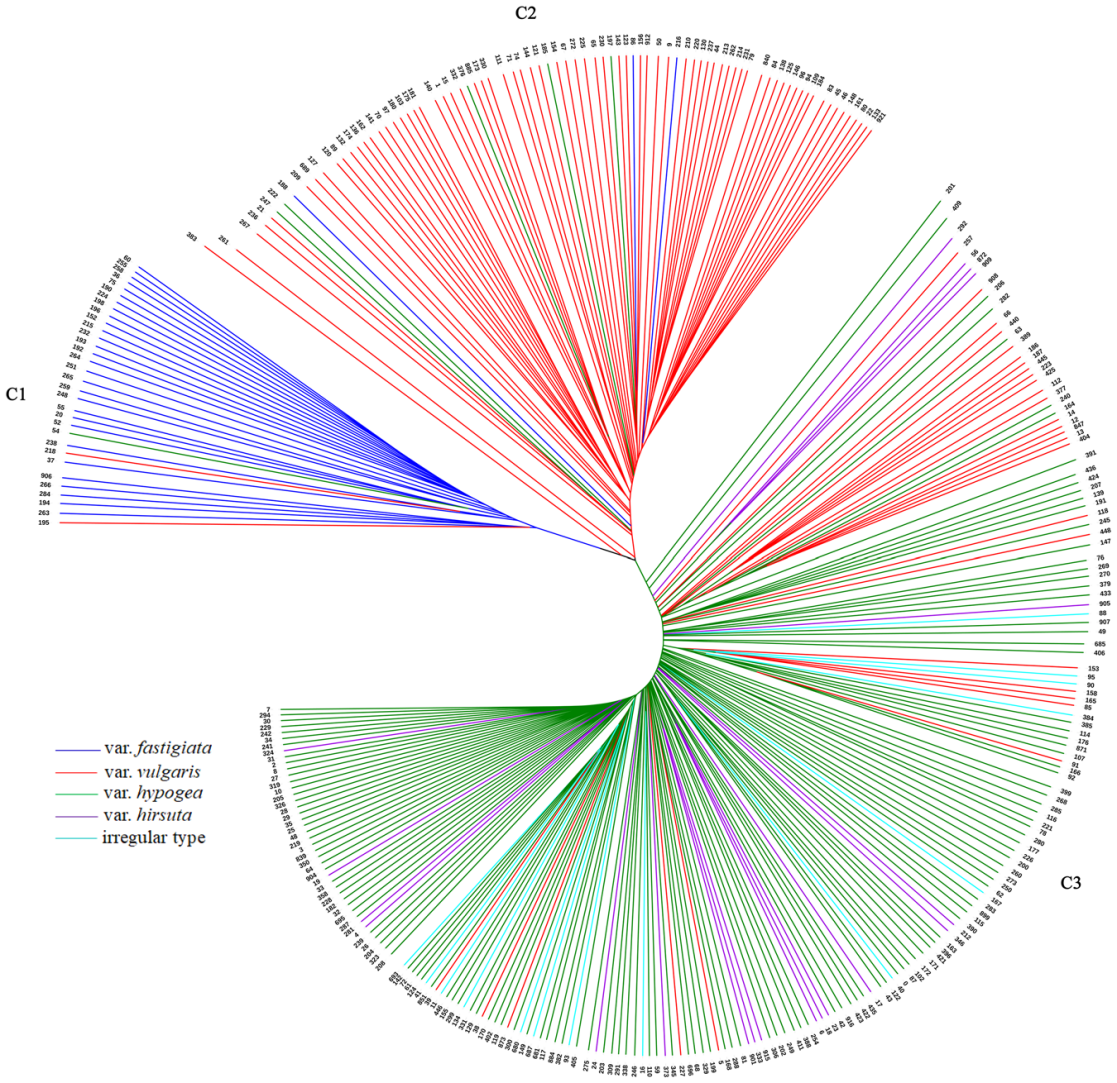
Phylogenetic tree of 320 peanut accessions constructed by UPGMA algorithm implemented in software TASSEL5.2.13 using 37,128 SNPs obtained from tGBS. The five botanical varieties var. *fastigiata*, var. *vulgaris*, var. *hypogaea*, var. *hirsuta*, and irregular type are colored in blue, red, green, purple and light green respectively. The 320 peanut accessions are clustered into three clusters C1, C2, and C3, which corresponding to var. *fastigiata*, var. *vulgaris*, and sub. *hypogaea* respectively.

A phylogenetic tree consisting of 320 peanut accessions was constructed based on the 37,128 high-quality SNPs obtained from tGBS (Fig. 2). The tested peanut accessions were clearly divided into three clusters (C1, C2, and C3). Clusters C1 and C2 mainly comprised var. *fastigiata* and var. *vulgaris*, respectively, from the subsp. *fastigiata*. Meanwhile, the accessions in cluster C3 included var. *hypogaea*, var. *hirsuta*, and irregular type, from the subsp. *hypogaea*.

Cluster C1 contained 32 peanut accessions, all of which were var. *fastigiata*, except for two accessions (Z195 and Z218), which were var. *vulgaris*, and one accession (Z54), which was var. *hypogaea*. Cluster C2 consisted of 81 peanut cultivars, which were var. *vulgaris*, with the exception of three accessions that were var. *fastigiata* and five cultivars that were var. *hypogaea*. The three var. *fastigiata* accessions (Z86, Z188, and Z216) were from the same subspecies as var. *vulgaris*. Four of the five var. *hypogaea* cultivars (Z185, Z197, Z222, and Z247) were from outside China, while the fifth cultivar (Z376) was a Chinese landrace. The common characteristic among these five cultivars was the production of a small pod (up to 25 mm long and 13 mm wide). Cluster C3 represented the largest cluster, with 144 var. *hypogaea*, 20 var. *hirsuta*, 29 var. *vulgaris*, and 14 irregular type accessions. Among the 29 var. *vulgaris* accessions, four (Z5, Z38, Z66, and Z908) were Chinese landraces, five (Z186, Z187, Z223, Z227, and Z257) were from outside China, and the remaining 20 accessions were Chinese breeding lines. Although these accessions were classified as var. *vulgaris*, their pods were larger than those of cluster C2 accessions. The 20 var. *hirsuta* and 14 irregular type peanut accessions were sparsely distributed in cluster C3 (Fig. 2).

### Principal component analysis

**Figure 3:**
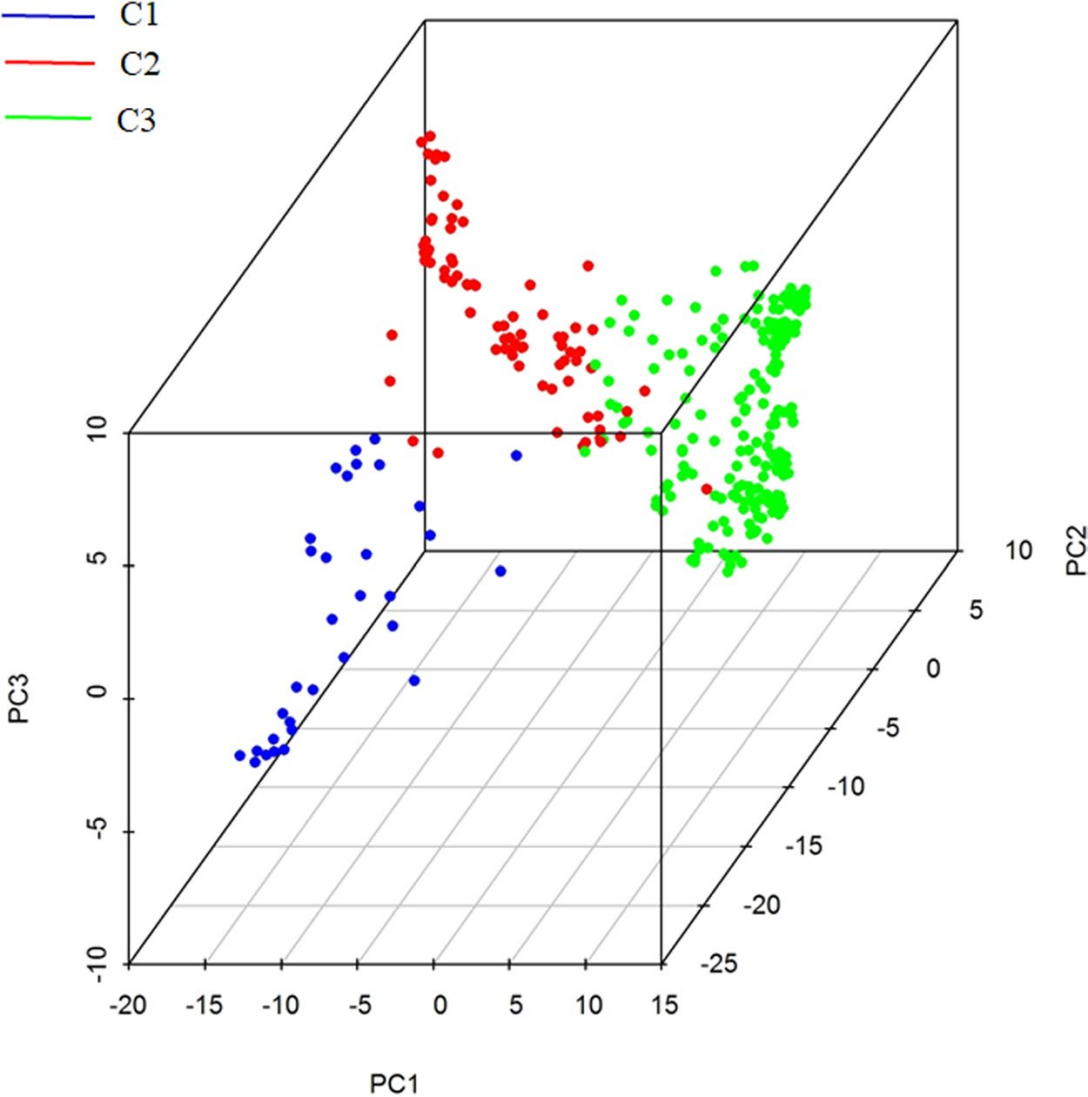
Three-dimensional scatter plots of the first three principal components. The blue dots stand for the first cluster (C1) in phylogenetic tree, consisted by varieties from var. *fastigiata*. The red dots display the distribution of the second cluster C2, mainly included germplasms from var. *vulgaris*. The green dots represent the cluster C3, most cultivars of which are from subsp. *hypogaea*.

Principal component analysis refers to a dimensionality reduction technique that is often applied to analyse genotype data to infer the population structures of genetic resources^25,26^. To further verify the clustering observed in the phylogenetic tree, a PCA was conducted using the same samples and SNP set. The scatter plots of the first three principal components were displayed in a three-dimensional space (Fig. 3). The scatter plots clearly revealed that the accessions in the three clusters of the phylogenetic tree were grouped separately, except for the slight overlap between clusters C2 and C3 (Fig. 3).

### Population structure analysis

**Figure 4:**
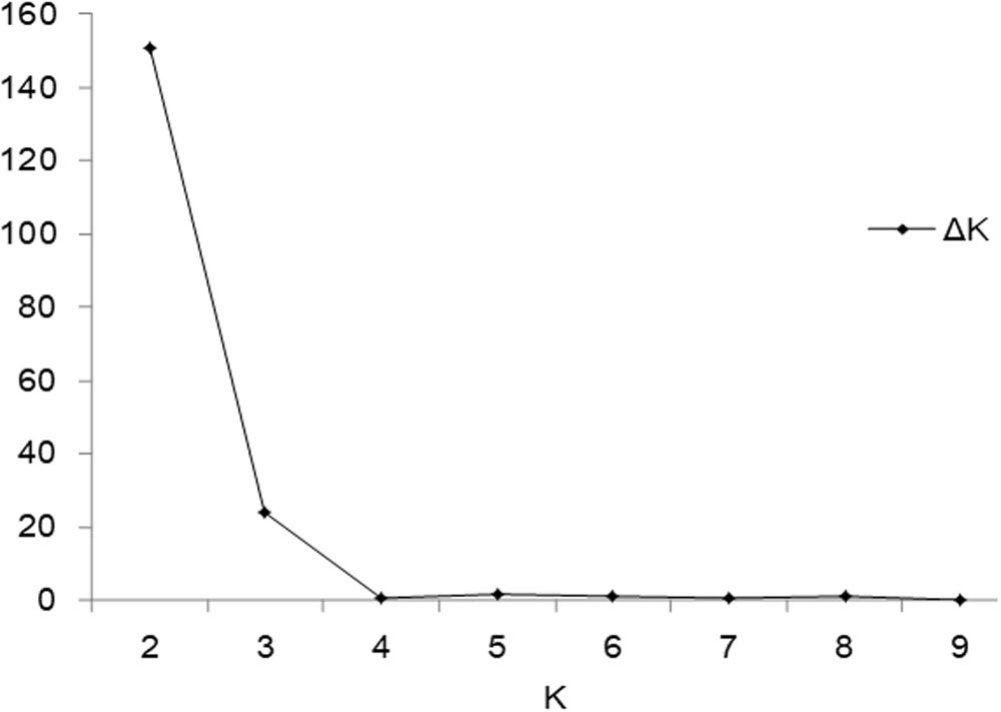
∆K based on the rate of change of LnP(D) between successive K. The value of Δ*K* reached the highest (150.957) when K = 2, indicating that 320 peanut accessions should be divided into two subgroups ∆*K* and reached the second largest value (23.720) with K = 3.

**Figure 5:**
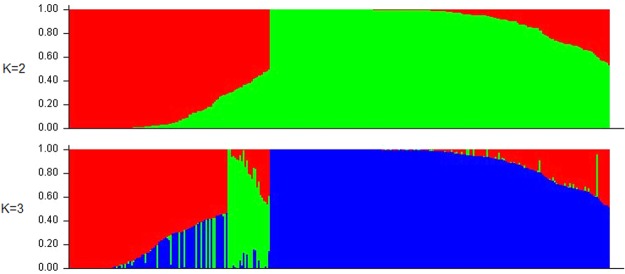
Structure analysis of subdivision of the population (K = 2 and 3) with STRUTURE 2.3.4. With K = 2, 320 peanut accession were divided into two subspecies, subsp. *fastigiata* and subsp. *hypogaea*. With K = 3, subsp. *fastigiata* was differentiated into two groups, corresponding to var. *vulgaris* and var. *fastigiata* respectively.

The population structure was explored with 37,128 SNPs and STRUCTURE v2.3.4^27^. Additionally, Structure Harverster^28^ was used to determine the optimal K value according to the maximum ΔK value. The highest ΔK value (150.957) was observed when K = 2 (Fig. 4). This result indicated that the 320 peanut accessions should be divided into two subgroups (G1 and G2), corresponding to two subspecies (sub. *fastigiata* and sub. *hypogaea*, respectively) (Fig. 5). The three subgroups (SG1, SG2, and SG3) when K = 3 (ΔK = 23.720) corresponded to var. *vulgaris*, var. *fastigiata*, and subsp. *hypogaea*, respectively. Moreover, when K = 3, var. *fastigiata* and var. *vulgaris* were clearly divided into two groups, whereas they were not in separate groups when K = 2 (Fig. 5).

As presented in Supplementary Table S3, when K = 2, the lines in clusters C1 and C2 were grouped in subgroup G1, except for accession Z383, which is a released variety from China. Meanwhile, the lines in cluster C3 belonged to subgroup G2, with the exception of seven Chinese breeding lines (Z112, Z377, Z164, Z12, Z847, Z13, and Z404). When K = 3, clusters C1, C2, and C3 in the evolutionary tree corresponded to subgroups SG2, SG1, and SG3, respectively. Additionally, consistent with the observations when K = 2, accession Z383 in cluster C2 was classified in subgroup SG3, while the same seven Chinese breeding lines (Z112, Z377, Z164, Z12, Z847, Z13, and Z404) in cluster C3 were grouped together in subgroup SG1 (i.e., botanical type of these closely related lines: var. *vulgaris*) (Supplementary Fig. 4). Moreover, another seven germplasms (Z195, Z263, Z194, Z284, Z266, Z218, and Z238) in cluster C1 were classified in subgroup SG1 when K = 3 (Supplementary Table S3). Among this group of germplasms, accessions Z195 and Z218 were var. *vulgaris*, while the other five accessions belonged to the same subspecies as var. *vulgaris*.

### Pairwise fixation index (*F*_*ST*_) and nucleotide diversity (π)

The *F*_*ST*_ and π values were used to determine the genetic differences among the three clusters in the evolutionary tree and the mean diversity for each cluster (Table 2). It should be noted that only landraces and the SNPs with a MAF greater than 0.05 and distributed on all 20 chromosomes (i.e., 9,378 SNPs) were used to calculate the *F*_*ST*_ value. This was because these landraces were not associated with any artificial hybridisations and were useful for elucidating the characteristics of different botanical types. Regarding the π value, all 320 peanut accessions and the 33,997 SNPs distributed on 20 peanut chromosomes (Supplementary Fig. S1) were used to calculate the mean diversity for each group.

**Table 2:** Pairwise *F*_*ST*_ between three clusters of phylogenetic tree (upper triangle) and the mean diversity (π) for each cluster (diagonal).

Cluster	C1	C2	C3
C1	0.035	0.284	0.452
C2		0.048	0.406
C3			0.012

The values in the upper triangle of Table 2 represent the *F*_*ST*_ values among the three clusters (C1, C2, and C3) in Fig. 2, while the diagonal values correspond to the π of each cluster. As expected, clusters C1 and C2 in the same subspecies had a closer relationship (0.284) than these clusters in different subspecies (0.452 and 0.406), indicating that var. *hypogaea* was clearly distinct from the other two botanical varieties (*F*_*ST*_ > 0.4). Similarly, the *F*_*ST*_ value between clusters C2 and C3 was smaller than that between clusters C1 and C3. The π values indicated that cluster C2 (0.048) had the broadest diversity, followed by clusters C1 (0.035) and C3 (0.012).

## Discussion

The genetic diversity and population structure of the 320 accessions were analysed using 37,128 high-quality SNPs obtained by tGBS. A phylogenetic analysis indicated the 320 peanut accessions were classified into clusters C1, C2, and C3, which corresponded to var. *fastigiata*, var. *vulgaris*, and subsp. *hypogaea*, respectively. To the best of our knowledge, this study is the first to reveal the genetic relationship between var. *fastigiata* and var. *vulgaris*. Moreover, the clusters were further validated by a PCA and a population structure analysis, with results that were consistent with the relationships indicated by the phylogenetic tree. Additionally, the *F*_*ST*_ values suggested that the varieties in the same subspecies (clusters C1 and C2) were more closely related than the varieties in different subspecies (clusters C1 and C3 as well as C2 and C3). Furthermore, the π values implied that cluster C2 had the broadest genetic diversity.

In this study, the botanical types of 320 peanut germplasms were first determined based on published information. The classifications of some accessions that exhibited characteristics that obviously contradicted their documented type were corrected, and accessions with no available information in published material were classified based on the following parameters: presence of flowers on the main stem, number of seeds per pod, pod appearance and shape, total number of branches, and plant growth type. However, these traits are very likely controlled by a few genetic factors and make the classification difficult for peanut breeders and researchers during their works and studies, especially when some of the accessions harbor mixed morphological features.

There were three types of revised classifications (Supplementary Table S3). First, peanut accessions with an undefined botanical type were classified. This group comprised 67 samples, including 15 Chinese landraces, 21 accessions from outside China, and 31 Chinese breeding lines. For example, the botanical type of Z198 was not determined by Belamkar *et al*.^4^, but the observation that Z198 plants contained flowers on the main stem and produced three seeds per pod suggested the botanical type of accession Z198 was var. *fastigiata*. Second, the botanical type of 17 varieties that were considered to be imperfectly defined in the available literature was revised. Although the botanical types of these accessions were based on information in published articles and databases, we believe the accessions were erroneously defined. These accessions were grouped in the right subspecies, but the incorrect botanical types were revised according to the number of seeds per pod or pod appearance. For example, Belamkar *et al*.^4^ determined that Z225 was var. *fastigiata*, but we revised this classification to var. *vulgaris* because the plants produced only two seeds per pod. Third, the botanical types of eight cultivars were revised between subspecies. For example, accession Z0, which is a landrace and a parental line of most Chinese breeding lines, was previously designated as var. *vulgaris*. However, we observed that this accession lacked flowers on the main stem, and produced relatively large seed pods with deep constrictions. Accordingly, we redefined accession Z0 as var. *hypogaea*.

The genetic diversity and population structure of 320 peanut accessions were evaluated with 37,128 high-quality SNPs. This study involved more markers and a larger sample size than most of the previous related studies, in which only a few SSR markers and peanut germplasms were used to investigate evolutionary relationships. For example, 19 SSR markers were used to analyse the genetic architecture of 24 peanut germplasms^29^, 34 SSR primer pairs were applied to evaluate the genetic diversity among 96 peanut cultivars^30^, and 14 SSR markers were used to investigate the genetic relationships among 90 released varieties in Henan province, China^31^. Although SSR markers have been extensively used to study peanuts^11^, their polymorphism rate is quite low and methods involving these markers are time-consuming and labour-intensive^8^. Thus, SNP markers represent a better option with the development of next-generation sequencing technology. A large SNP dataset was recently used for genetic assessments for a genome-wide association study (GWAS) and the development of a SNP array^9,10^.

In this study, var. *fastigiata* and var. *vulgaris* in subsp. *fastigiata* were distinguished for the first time. The 320 peanut accessions were grouped into three clusters according to the phylogenetic tree, with the first two clusters consisting of var. *fastigiata* and var. *vulgaris* accessions (subsp. *fastigiata*), while the third cluster comprised var. *hypogaea*, var. *hirsuta*, and irregular type accessions (subsp. *hypogaea*). Moreover, var. *fastigiata* and var. *vulgaris* in subsp. *fastigiata* were clearly divided into two clusters (Fig. 2), while the var. *hirsuta* samples were scattered among var. *hypogaea* samples. In all other related studies^4,8,9^, only two subspecies (*fastigiata* and *hypogaea*) were clearly distinguished. The two most recent SNP-based genetic analyses of peanuts included 17,338 SNPs developed by SLAF-seq.^9^ and a 58 K SNP array^10^. Both of these studies were only able to confirm the relationship between two subspecies. Further, the 14 irregular type accessions were scattered only in the subsp. *hypogaea* in this study. These accessions were all released varieties derived from crossing between the two subsp. in northern China, where the subsp. *hypogaea* were the popular botanical type. It was implied that the released irregular types were more similar to the subsp. *hypogaea* in phenotype under the breeding selection and possessed great proportion of genetic background from subsp. *hypogaea*, thus, they were classified to subsp. *hypogaea* subsequently. Therefore, when a peanut accession with a complex mixture of genetic background is difficult to be assigned to subspecies like the mentioned “irregular types”, genotyping with GBS or high density molecular markers could be applied as a good solution. Our data revealed that the genetic distance of the tested peanut accessions ranged from 0.043 to 0.127, with an average of 0.080, while a previous genetic distance ranged from 0.019 to 0.837 with an average of 0.468 was reported by Zhang *et al*.^32^. The smaller range in our case is likely because our study included more marker loci taking account that the diverse samples were carefully selected from over 2,000 germplasms. The largest average genetic distance was observed for var. *vulgaris*, followed by var. *fastigiata*, var. *hypogaea*, var. *hirsuta*, and irregular type. Therefore, genetic diversity was greatest for var. *vulgaris*, followed by var. *fastigiata* and var. *hypogaea*, which was consistent with the observed π.

A pseudo-reference genome was developed based on the genomes of two diploid progenitors of cultivated peanut, *A*. *duranensis* (A genome) and *A*. *ipaensis* (B genome). To identify unique read loci, genomes A and B were artificially merged to form 20 chromosomes, which were then used for SNP calling. This may have resulted in some inaccuracies in the read mapping and detection of SNP loci. However, this method represents the only viable option, at least until the tetraploid *A*. *hypogaea* L. genome is published. A set of 37,128 high-quality SNPs, with a 30% missing data rate, was generated through tGBS. These SNPs were obtained based on the requirement that the minor allele is present in at least three samples and at least one homozygous sample (i.e., MAF of approximately 0.01). When the MAF was increased to 0.05, only 10,004 SNPs remained. However, the same three clusters were included in the phylogenetic tree regardless of whether 10,004 SNPs or 37,128 SNPs were used (data not shown).To verify our classification results, we analysed the pedigrees of nine accessions (Z112, Z377, Z240, Z164, Z14, Z12, Z847, Z13, and Z404) that were grouped together in a branch of cluster C3 in the phylogenetic tree (Fig. S4). It indicated that eight of these nine accessions are closely related except for Z240, which is an accession collected from the germplasm bank and its pedigree information is not available. Further effort is needed to investigate its genetic background.

There are additional minor problems associated with SNP calling and genetic analyses of peanut accessions. First, although the missing data rate for each SNP across 320 samples was maintained within 30%, the missing data rate for all SNPs in each sample was neglected in most studies, which may have resulted in errors. For example, the abnormal position of accession Z383 in the phylogenetic tree may be due to its high missing data rate (55.1%). This accession is a released var. *vulgaris* cultivar, which should be in cluster C3, at least according to the population structure analyses when K = 2 or 3 (Supplementary Table S3). Second, the LD decay plots were not satisfactorily produced based on 37,128 SNPs, possibly because of a limited number of SNPs and/or an uneven distribution of SNPs on the 20 chromosomes. Hence, whole genome resequencing with high coverage and a reliable cultivated peanut reference genome represents a better option for investigating allotetraploid cultivated peanut crops with abundant repetitive sequences.

## Materials and Methods

### Plant materials

A total of 320 peanut accessions were selected from the Henan Academy of Agricultural Sciences germplasm resource, which comprises about 2,000 peanut germplasms from around the world. The accessions were collected based on phenotypic characteristics, including plant height, number of total branches, and growth habit (spreading or erect), as well as the results of a cluster analysis of molecular markers (Supplementary Table S3). The selected peanut accessions included 100 landraces and 133 breeding lines from China and 87 accessions from the US mini-core collection^4^. The botanical information for the selected Chinese varieties was derived from available monographs^33–36^ and an online database (http://www.peanutdata.cn). Details regarding the remaining varieties were obtained from published articles^4,8,37–41^. Additionally, all selected peanut accessions were grown at the experimental station of the Henan Academy of Agricultural Sciences, Yuanyang, Henan, China for a subsequent morphological examination. A total of 67 accessions lacking available information were evaluated and the classifications for 25 accessions were revised according to the presence of flowers on the main stem, number of seeds per pod, number of branches, pod appearance, and growth habit (spreading or erect) according to the descriptions for different botanical varieties^4^. The 320 peanut accessions consisted of 150 var. *hypogaea*, 104 var. *vulgaris*, 32 var. *fastigiata*, 20 var. *hirsuta*, and 14 irregular type accessions.

### DNA extraction and sequencing

The QIAGEN DNeasy Plant Mini Kit was used to extract genomic DNA from the fresh leaves of a single plant for each accession. The DNA concentrations for the 320 samples were measured with the NanoDrop-2000 spectrophotometer. For each accession, approximately 20 µg DNA was transferred to 96-well plates and freeze-dried for tGBS, which was completed by Data2Bio (USA). The DNA samples were genotyped using an Illumina HiSeq. 2000 system and the Data2Bio proprietary tGBS technology^17^.

### SNP calling

The reference genomes of two diploid progenitors of cultivated peanut (Aradu_v1.0.fa and Araip_v1.0.fa) were obtained from peanutbase.org and used as the pseudo-reference, with 2,059 contigs and a total length of 2.44 Gb^2^. Each sequenced read was scanned for low-quality regions and bases. Reads with a PHRED quality score less than 15 out of 40 (i.e., error rate ≤ 3%) were trimmed^21,22^. The trimmed reads for each sample were aligned to the reference genome using the GSNAP aligner^23^. Reads corresponding to the best alignments (i.e., maximum of two mismatches for every 36 bp of read length and a maximum of 5 bp for every 75 bp of tail read length) were then extracted and used to identify SNPs. On average, each SNP was supported by nine reads, ensuring accurate genotyping.

### Analysis of genotyping data

The genetic distance matrix was calculated, an unweighted pair group method with arithmetic average cladogram^42^ was constructed and the principal component analysis (PCA) was completed using TASSEL5.2.13 (http://www.maizegenetics.net/tassel)^24^.The archaeopteryx tree was saved in the Newick format and a phylogenetic tree was prepared using the iTOL v4 online tool (http://itol.embl.de/)^43^. A three-dimensional scatter plot of the first three principal components was plotted using the R package “scatterplot3d” (https://cran.r-project.org/web/packages/scatterplot3d/index.html)^44^.

A model-based (Bayesian) clustering method implemented with STRUCTURE v2.3.4 (http://web.stanford.edu/group/pritchardlab/structure.html)^27^ was used to investigate the population structure. The program was run 10 times for each K value, ranging from 1 to 10, with a 1,000 burn-in time and 1,000 iterations. The optimal K value was determined based on the ΔK from the Structure Harvester v0.6.94 (http://alumni.soe.ucsc.edu/~dearl/software/structureHarvester/)^28^ program.

The pairwise fixation index (*F*_*ST*_), which explains the genetic differentiation between sub-populations, was estimated with Genepop v4.6 (http://kimura.univ-montp2.fr/%7Erousset/Genepop.htm)^45^. Data for the *F*_*ST*_ analysis were pre-processed by keeping SNPs with a minor allele frequency (MAF) greater than 0.05 and removing SNPs from scaffolds. To eliminate the influence of genetic crosses, only landraces were selected for the *F*_*ST*_ analysis.

Nucleotide diversity (π) was assessed using MEGA v7.0 (https://www.megasoftware.net/)^46^, with 300 bootstraps and the maximum composite likelihood model. Nucleotide diversity is a molecular genetics concept used to measure the degree of polymorphism within a population^47^. The SNP data for scaffolds were removed before calculating the mean diversity for each group.

## Electronic supplementary material

Supplementary information for tGBS analysis of 320 peanut accessions

## Data Availability

The raw data of this study has been deposited in the NCBI Sequence Read Archive (SRA) under accession SRP152747.
