# Supplementary material for: Genetic Diversity, Population Structure, and Botanical Variety of 320 Global Peanut Accessions Revealed Through Tunable Genotyping-by-Sequencing
Source: Sci Rep. 2018 Sep 28;8:14500. doi: 10.1038/s41598-018-32800-9 (PMC6162295; doi:10.1038/s41598-018-32800-9)
Supplement: Supplementary file 1 — Supplementary information for tGBS analysis of 320 peanut accessions [file 41598_2018_32800_MOESM1_ESM.pdf]

Genetic Diversity, Population Structure, and Botanical Variety of 320 Global Peanut Accessions Revealed Through Tunable Genotyping-by-Sequencing

Zheng Zheng[1#], Ziqi Sun[1#], Yuanjin Fang[1], Feiyan Qi[1], Hua Liu[1], Lijuan Miao[1], Pei Du[1], Lei Shi[1], Wei Gao[1], Suoyi Han[1],Wenzhao Dong[1], Fengshou Tang[1], Feng Cheng[2], Haiyan Hu[1], Bingyan Huang[1*], Xinyou Zhang[1*]

[1]Industrial Crops Research Institute, Henan Academy of Agricultural Sciences / Key Laboratory of Oil Crops in Huanghuaihai Plains, Ministry of Agriculture / Henan Provincial Key Laboratory for Genetic Improvement of Oil Crops, Zhengzhou, 450002, China;

[2]Institute of Vegetables and Flowers, Chinese Academy of Agricultural Science, Beijing, 100081, China

Corresponding authors: Xinyou Zhang and Bingyan Huang

Address: No. 116 Huayuan Road, Jinshui District, Zhengzhou 450002, China

Telephone numbers: +86 0371-65729560, +86 0371-65718247

Fax numbers: +86 0371-65739143

Email: haasz@126.com, huangbingyan@aliyun.com

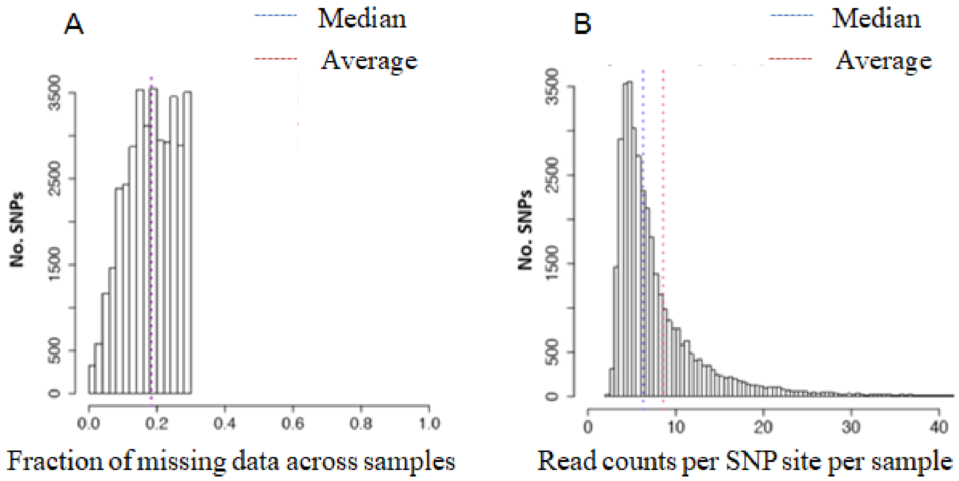

Supplementary Figure 1 Histograms of average missing data rate and average number of reads per SNP site

(A) Histogram of average missing data rate of the 37,128 SNPs across 320 peanut samples. The average missing data rate is 18.1% and the median is 18.4%. (B) Histogram of read counts of 37,128 SNP sites for each peanut accession. On average, each SNP call in each sample was supported by 9 reads and the median is 6 reads per SNP site per sample.

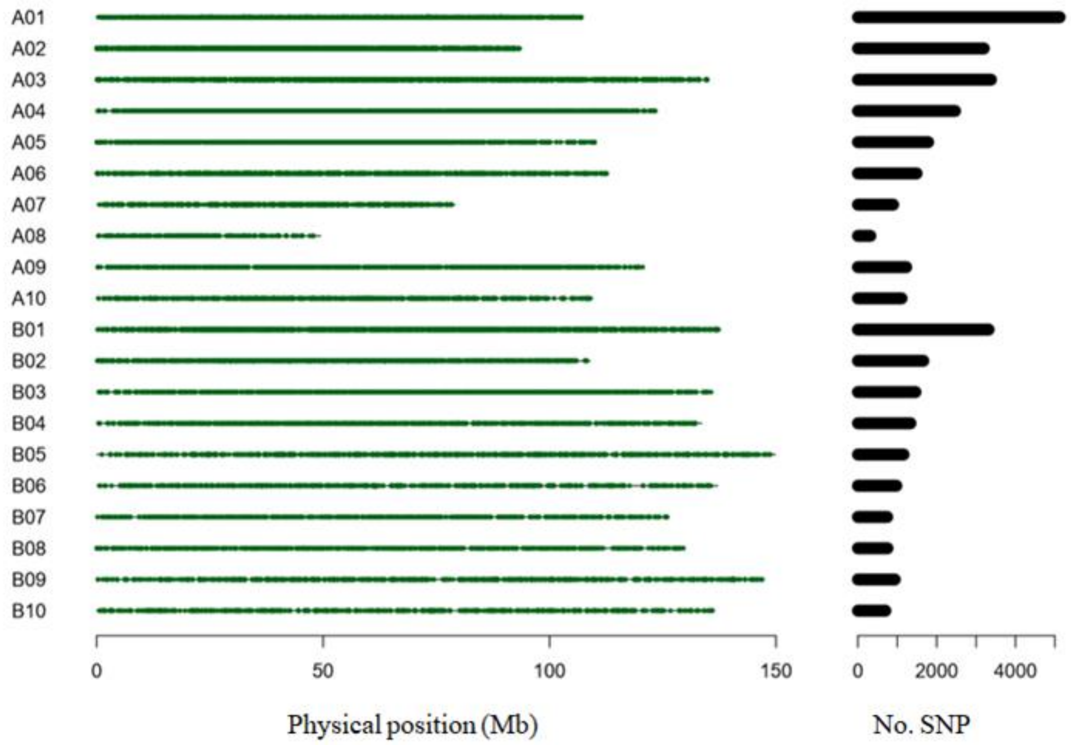

Supplementary Figure 2 Distribution of 33,997 SNPs and the number on each chromosome

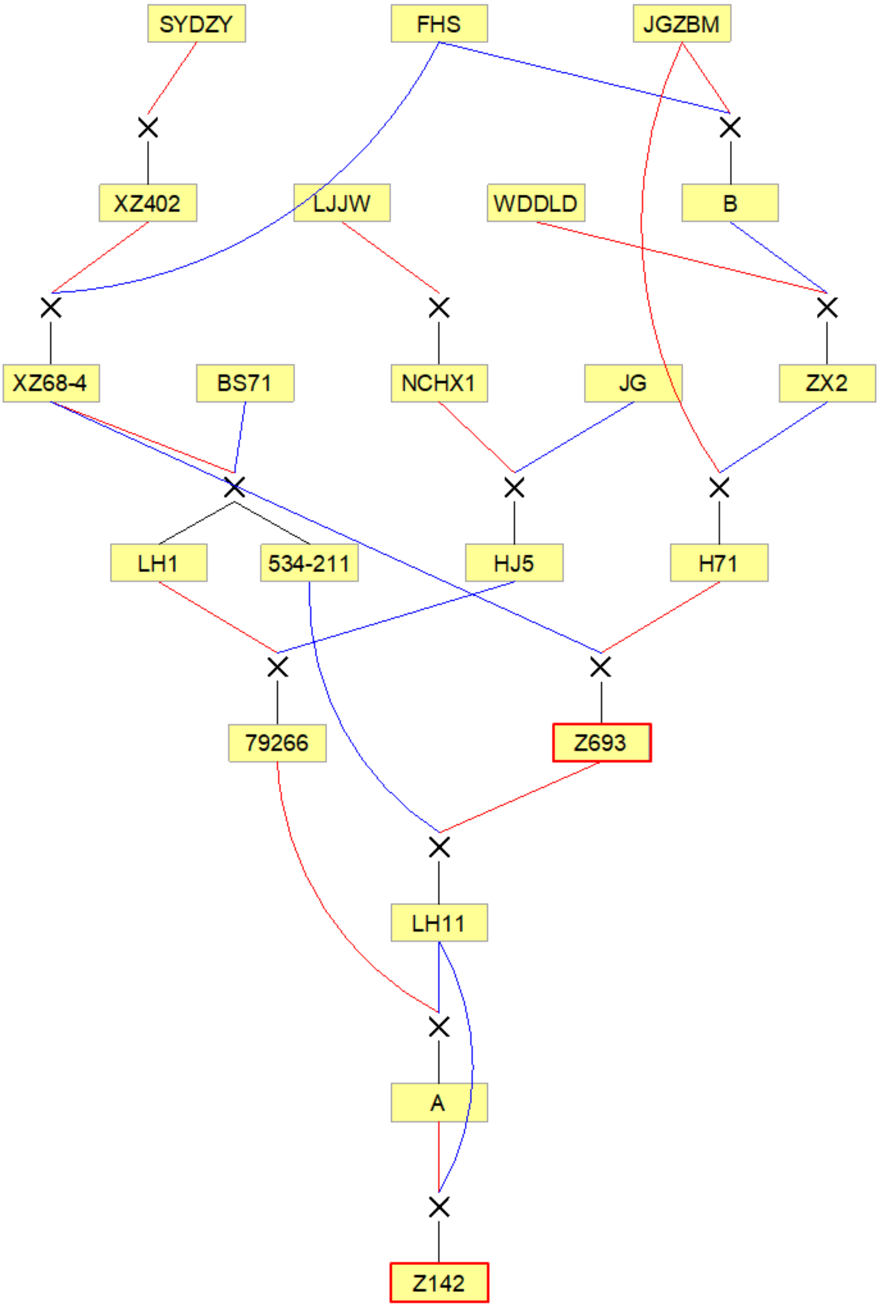

Supplementary Figure 3 Pedigree information of peanut accessions Z142 and Z693

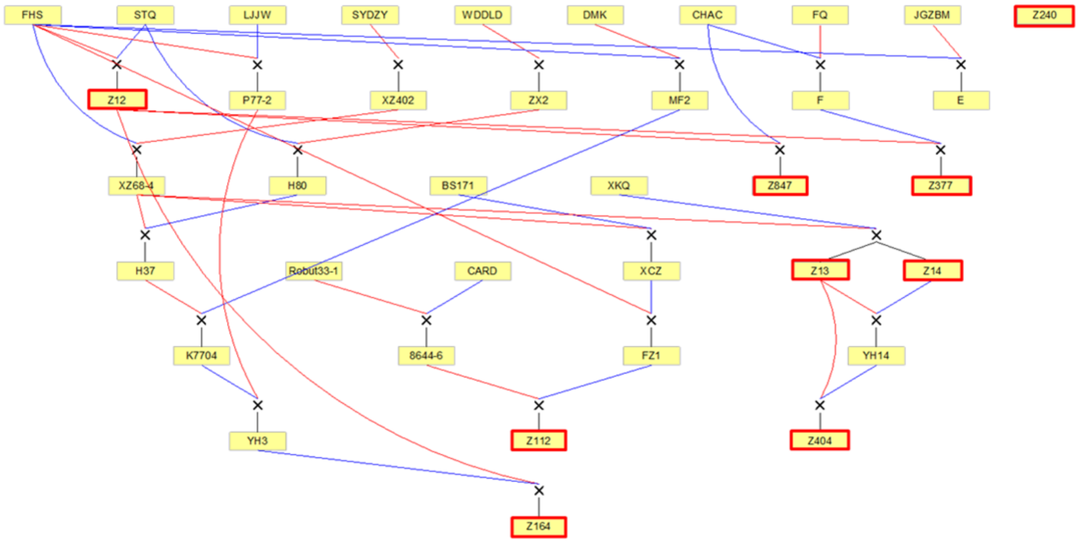

Supplementary Figure 4 Pedigree information of 9 peanut accessions (Z112, Z377, Z240, Z164, Z14, Z12, Z847, Z13 and Z404)

Supplementary Table S1 Distribution and density of SNPs identified by tGBS in this study

Chromosome	SNP numbers	Chromosome size (bp)*	SNP density (per 100kb)
Aradu.A01	5,119	106,015,837	4.83
Aradu.A02	3,195	92,627,924	3.45
Aradu.A03	3,376	133,136,946	2.54
Aradu.A04	2,465	121,180,382	2.03
Aradu.A05	1,781	108,284,737	1.64
Aradu.A06	1,490	110,733,117	1.35
Aradu.A07	894	77,948,624	1.15
Aradu.A08	315	48,937,534	0.64
Aradu.A09	1,225	118,999,574	1.03
Aradu.A10	1,107	107,255,536	1.03
Araip.B01	3,317	136,910,013	2.42
Araip.B02	1,660	108,641,379	1.53
Araip.B03	1,460	135,595,063	1.08
Araip.B04	1,333	133,209,281	1.00
Araip.B05	1,158	149,435,236	0.77
Araip.B06	976	136,721,448	0.71
Araip.B07	743	125,994,751	0.59
Araip.B08	750	129,151,520	0.58
Araip.B09	936	146,495,397	0.64
Araip.B10	697	135,809,342	0.51
Total	33,997	2,363,083,641	1.44

*Chromosome size was obtained from: Nature Genetics,(2016).48(4), 438-446. DOI: 10.1038/ng.3517

Supplementary Table S2 Distance matrix for genotypes of the Reference set using polymorphic SNP markers

Table S2 Distance matrix for genotypes of the Reference set using polymorphic SNP markers

[illegible]	[illegible]

Supplementary Table S3 The details of 320 peanut accessions used in genetic analysis

Accession	Type	Country	Clusters in Phylogenetic Tree	Structure _K=2	Structure _K=3	Botainical variety after checked
Z0	Landrace	China	C3	G2	G3	var. hypogaea[3]
Z1	Landrace	China	C2	G1	G1	var. vulgaris
Z2	Landrace	China	C3	G2	G3	var. hypogaea
Z3	Landrace	China	C3	G2	G3	var. hypogaea
Z4	Landrace	China	C3	G2	G3	var. hirsuta
Z5	Landrace	China	C3	G2	G3	var. vulgaris
Z6	Landrace	China	C3	G2	G3	var. hirsuta
Z7	Landrace	China	C3	G2	G3	var. hypogaea[1]
Z8	Landrace	China	C3	G2	G3	var. hypogaea
Z9	Landrace	China	C2	G1	G1	var. vulgaris
Z10	Landrace	China	C3	G2	G3	var. hypogaea
Z11	Breeding Line	China	C3	G2	G3	irregular type
Z12	Breeding Line	China	C3	G1	G1	var. vulgaris
Z13	Breeding Line	China	C3	G1	G1	var. vulgaris
Z14	Breeding Line	China	C3	G2	G3	var. vulgaris[1]
Z15	Breeding Line	China	C2	G1	G1	var. vulgaris
Z16	Breeding Line	China	C3	G2	G3	irregular type
Z17	Breeding Line	China	C3	G2	G3	var. hirsuta
Z18	Breeding Line	China	C3	G2	G3	var. hirsuta
Z19	Landrace	China	C3	G2	G3	var. hirsuta
Z20	Landrace	China	C1	G1	G2	var. fastigiata
Z21	Landrace	China	C2	G1	G1	var. vulgaris
Z22	Landrace	China	C2	G1	G1	var. vulgaris
Z23	Landrace	China	C3	G2	G3	var. hirsuta
Z24	Landrace	China	C3	G2	G3	var. hirsuta

Z25	Landrace	China	C3	G2	G3	var. hypogaea
Z26	Landrace	China	C3	G2	G3	var. hypogaea
Z27	Landrace	China	C3	G2	G3	var. hypogaea
Z28	Landrace	China	C3	G2	G3	var. hypogaea
Z29	Landrace	China	C3	G2	G3	var. hypogaea
Z30	Landrace	China	C3	G2	G3	var. hypogaea
Z31	Landrace	China	C3	G2	G3	var. hypogaea
Z32	Landrace	China	C3	G2	G3	var. hypogaea
Z33	Landrace	China	C3	G2	G3	var. hypogaea
Z34	Landrace	China	C3	G2	G3	var. hypogaea
Z35	Landrace	China	C3	G2	G3	var. hypogaea
Z36	Landrace	China	C1	G1	G2	var. fastigiata
Z37	Landrace	China	C1	G1	G2	var. fastigiata
Z38	Landrace	China	C3	G2	G3	var. vulgaris
Z39	Breeding Line	China	C3	G2	G3	var. vulgaris
Z40	Breeding Line	China	C3	G2	G3	irregular type
Z41	Breeding Line	China	C3	G2	G3	irregular type
Z42	Breeding Line	China	C3	G2	G3	var. hypogaea
Z43	Breeding Line	China	C3	G2	G3	var. hypogaea
Z44	Landrace	China	C2	G1	G1	var. vulgaris
Z45	Breeding Line	China	C2	G1	G1	var. vulgaris
Z46	Breeding Line	China	C2	G1	G1	var. vulgaris
Z48	Landrace	China	C3	G2	G3	var. hypogaea[3]
Z49	Breeding Line	China	C3	G2	G3	var. hypogaea[3]
Z50	Landrace	China	C2	G1	G1	var. vulgaris
Z52	Landrace	China	C1	G1	G2	var. fastigiata[3]
Z54	Landrace	China	C1	G1	G2	var. hypogaea
Z55	Landrace	China	C1	G1	G2	var. fastigiata[3]

Z56	Landrace	China	C3	G2	G3	var. hirsuta
Z59	Breeding Line	China	C3	G2	G3	var. hypogaea[1]
Z60	Landrace	China	C1	G1	G2	var. fastigiata
Z61	Landrace	China	C3	G2	G3	var. hypogaea
Z62	Landrace	China	C3	G2	G3	var. hypogaea
Z63	Landrace	China	C3	G2	G3	var. hypogaea[1]
Z64	Landrace	China	C3	G2	G3	var. hypogaea[1]
Z65	Landrace	China	C2	G1	G1	var. vulgaris
Z66	Landrace	China	C3	G2	G3	var. vulgaris
Z67	Landrace	China	C2	G1	G1	var. vulgaris
Z68	Landrace	China	C3	G2	G3	var. hypogaea
Z70	Breeding Line	China	C2	G1	G1	var. vulgaris
Z71	Breeding Line	USA	C2	G1	G1	var. vulgaris
Z72	Overseas	India	C3	G2	G3	var. hypogaea
Z74	Overseas	USA	C2	G1	G1	var. vulgaris
Z75	Overseas	USA	C1	G1	G2	var. fastigiata
Z76	Overseas	ND	C3	G2	G3	var. hypogaea[1]
Z78	Overseas	ND	C3	G2	G3	var. hypogaea[1]
Z79	Overseas	Senegal	C2	G1	G1	var. vulgaris[1]
Z80	Landrace	Senegal	C2	G1	G1	var. vulgaris
Z81	Overseas	Senegal	C3	G2	G3	var. hypogaea[1]
Z83	Breeding Line	China	C2	G1	G1	var. vulgaris
Z84	Breeding Line	China	C2	G1	G1	var. vulgaris
Z85	Breeding Line	China	C3	G2	G3	var. vulgaris
Z86	Landrace	China	C2	G1	G1	var. fastigiata
Z87	Breeding Line	China	C3	G2	G3	var. hypogaea[1]
Z88	Breeding Line	China	C3	G2	G3	irregular type

Z89	Landrace	China	C2	G1	G1	var. vulgaris
Z90	Breeding Line	China	C3	G2	G3	irregular type
Z91	Breeding Line	China	C3	G2	G3	var. vulgaris
Z92	Breeding Line	China	C3	G2	G3	var. hypogaea
Z93	Breeding Line	China	C3	G2	G3	irregular type
Z94	Breeding Line	China	C2	G1	G1	var. vulgaris[1]
Z95	Breeding Line	China	C3	G2	G3	irregular type
Z96	Breeding Line	China	C2	G1	G1	var. vulgaris
Z97	Breeding Line	China	C2	G1	G1	var. vulgaris
Z102	Breeding Line	China	C3	G2	G3	var. hypogaea[1]
Z103	Breeding Line	China	C2	G1	G1	var. vulgaris
Z107	Breeding Line	China	C3	G2	G3	var. hypogaea[1]
Z109	Breeding Line	China	C2	G1	G1	var. vulgaris[1]
Z110	Breeding Line	China	C3	G2	G3	var. hypogaea
Z111	Breeding Line	China	C2	G1	G1	var. vulgaris
Z112	Breeding Line	China	C3	G1	G1	var. vulgaris[1]
Z114	Breeding Line	China	C3	G2	G3	var. hypogaea[1]
Z115	Breeding Line	China	C3	G2	G3	var. hypogaea[1]
Z116	Breeding Line	China	C3	G2	G3	var. hypogaea
Z117	Breeding Line	China	C3	G2	G3	var. hypogaea
Z118	Breeding Line	China	C3	G2	G3	var. vulgaris
Z119	Breeding Line	China	C3	G2	G3	var. hypogaea
Z120	Landrace	China	C2	G1	G1	var. vulgaris
Z121	Breeding Line	China	C2	G1	G1	var. vulgaris
Z122	Breeding Line	China	C3	G2	G3	var. hypogaea[1]
Z123	Landrace	China	C2	G1	G1	var. vulgaris
Z124	Breeding Line	China	C3	G2	G3	var. hypogaea

Z125	Breeding Line	China	C2	G1	G1	var. vulgaris
Z127	Breeding Line	China	C2	G1	G1	var. vulgaris
Z129	Breeding Line	China	C3	G2	G3	var. hypogaea[1]
Z130	Breeding Line	China	C2	G1	G1	var. vulgaris[1]
Z132	Breeding Line	China	C2	G1	G1	var. vulgaris[1]
Z133	Landrace	China	C2	G1	G1	var. vulgaris
Z134	Breeding Line	China	C3	G2	G3	irregular type
Z136	Breeding Line	China	C2	G1	G1	var. vulgaris[1]
Z138	Breeding Line	China	C2	G1	G1	var. vulgaris
Z139	Breeding Line	China	C3	G2	G3	var. hypogaea
Z140	Breeding Line	China	C2	G1	G1	var. vulgaris
Z141	Breeding Line	China	C2	G1	G1	var. vulgaris
Z142	Breeding Line	China	C3	G2	G3	var. hypogaea
Z143	Breeding Line	China	C2	G1	G1	var. vulgaris
Z144	Breeding Line	China	C2	G1	G1	var. vulgaris
Z146	Breeding Line	China	C2	G1	G1	var. vulgaris
Z147	Breeding Line	China	C3	G2	G3	var. hypogaea
Z148	Landrace	China	C2	G1	G1	var. vulgaris[2]
Z149	Breeding Line	China	C3	G2	G3	var. hypogaea
Z152	Breeding Line	China	C1	G1	G2	var. fastigiata
Z153	Breeding Line	China	C3	G2	G3	var. vulgaris
Z154	Breeding Line	China	C2	G1	G1	var. vulgaris
Z155	Breeding Line	China	C3	G2	G3	var. hypogaea
Z156	Landrace	China	C2	G1	G1	var. vulgaris
Z158	Breeding Line	China	C3	G2	G3	var. vulgaris
Z161	Landrace	China	C2	G1	G1	var. vulgaris
Z162	Breeding Line	China	C2	G1	G1	var. vulgaris[1]
Z163	Breeding Line	China	C3	G2	G3	var. hypogaea

Z164	Breeding Line	China	C3	G1	G1	var. hypogaea[1]
Z165	Breeding Line	China	C3	G2	G3	var. vulgaris
Z166	Breeding Line	China	C3	G2	G3	var. hypogaea[1]
Z167	Breeding Line	China	C3	G2	G3	irregular type
Z168	Landrace	China	C3	G2	G3	var. hypogaea
Z170	Breeding Line	China	C3	G2	G3	var. hypogaea[1]
Z171	Breeding Line	China	C3	G2	G3	var. hypogaea[1]
Z172	Breeding Line	China	C3	G2	G3	var. hypogaea[1]
Z173	Breeding Line	China	C2	G1	G1	var. vulgaris[1]
Z174	Breeding Line	China	C2	G1	G1	var. vulgaris[1]
Z175	Breeding Line	China	C2	G1	G1	var. vulgaris
Z176	Breeding Line	China	C3	G2	G3	var. hypogaea[1]
Z177	Breeding Line	China	C3	G2	G3	var. hypogaea
Z180	Breeding Line	China	C2	G1	G1	var. vulgaris
Z181	Breeding Line	China	C2	G1	G1	var. vulgaris
Z182	Landrace	China	C3	G2	G3	var. hypogaea
Z184	Breeding Line	China	C2	G1	G1	var. vulgaris
Z185	Overseas	Mozambique	C2	G1	G1	var. hypogaea
Z186	Overseas	Nigeria	C3	G2	G3	var. vulgaris
Z187	Overseas	Zimbabwe	C3	G2	G3	var. vulgaris[2]
Z188	Overseas	Zimbabwe	C2	G1	G1	var. fastigiata
Z190	Overseas	Brazil	C1	G1	G2	var. fastigiata
Z191	Overseas	Paraguay	C3	G2	G3	var. hypogaea[3]
Z192	Overseas	Paraguay	C1	G1	G2	var. fastigiata
Z193	Overseas	Paraguay	C1	G1	G2	var. fastigiata
Z194	Overseas	Paraguay	C1	G1	G1	var. fastigiata[2]
Z195	Overseas	Paraguay	C1	G1	G1	var. vulgaris[2]

Z196	Overseas	Brazil	C1	G1	G2	var. fastigiata
Z197	Overseas	Burkina Faso	C2	G1	G1	var. hypogaea
Z198	Overseas	Argentina	C1	G1	G2	var. fastigiata[1]
Z199	Overseas	India	C3	G2	G3	var. hypogaea
Z200	Overseas	India	C3	G2	G3	var. hypogaea
Z201	Overseas	Israel	C3	G2	G2	var. hypogaea[1]
Z202	Overseas	Madagascar	C3	G2	G3	var. hypogaea
Z203	Overseas	Malawi	C3	G2	G3	var. hypogaea
Z204	Overseas	Mexico	C3	G2	G3	var. hypogaea
Z205	Overseas	Nigeria	C3	G2	G3	var. hypogaea
Z206	Overseas	Nigeria	C3	G2	G3	var. hypogaea
Z207	Overseas	Paraguay	C3	G2	G3	var. hypogaea
Z208	Overseas	Senegal	C3	G2	G3	var. hypogaea[3]
Z209	Overseas	South Africa	C2	G1	G1	var. vulgaris[1]
Z210	Overseas	South Africa	C2	G1	G1	var. vulgaris[1]
Z212	Overseas	Taiwan	C3	G2	G3	var. hirsuta[1]
Z213	Overseas	Uruguay	C2	G1	G1	var. vulgaris[2]
Z214	Overseas	Uruguay	C2	G1	G1	var. vulgaris[2]
Z215	Overseas	Brazil	C1	G1	G2	var. fastigiata
Z216	Overseas	Brazil	C2	G1	G1	var. fastigiata
Z218	Overseas	Zambia	C1	G1	G1	var. vulgaris
Z219	Overseas	Cuba	C3	G2	G3	var. hypogaea
Z220	Overseas	India	C2	G1	G1	var. vulgaris[1]
Z221	Overseas	Israel	C3	G2	G3	var. hypogaea[1]
Z222	Overseas	Venezuela	C2	G1	G1	var. hypogaea
Z223	Overseas	Zambia	C3	G2	G3	var. vulgaris[1]
Z224	Overseas	Argentina	C1	G1	G2	var. fastigiata[2]

Z225	Overseas	Uruguay	C2	G1	G1	var. vulgaris[2]
Z226	Overseas	China	C3	G2	G3	var. hypogaea
Z227	Overseas	China	C3	G2	G3	var. vulgaris[2]
Z228	Overseas	Sudan	C3	G2	G3	var. hypogaea
Z229	Overseas	Ivory Coast	C3	G2	G3	var. hypogaea
Z230	Overseas	Japan	C2	G1	G1	var. vulgaris
Z231	Overseas	South Africa	C2	G1	G1	var. vulgaris[2]
Z232	Overseas	Cuba	C1	G1	G2	var. fastigiata
Z236	Overseas	Zambia	C2	G1	G1	var. vulgaris[1]
Z237	Overseas	Zambia	C2	G1	G1	var. vulgaris[2]
Z238	Overseas	India	C1	G1	G1	var. fastigiata[2]
Z239	Overseas	India	C3	G2	G3	var. hypogaea
Z240	Overseas	Argentina	C3	G2	G3	var. hypogaea
Z241	Overseas	South Africa	C3	G2	G3	var. hypogaea
Z242	Overseas	Israel	C3	G2	G3	var. hypogaea
Z245	Overseas	Israel	C3	G2	G3	var. hypogaea
Z246	Overseas	Pakistan	C3	G2	G3	var. hypogaea
Z247	Overseas	Morocco	C2	G1	G1	var. hypogaea
Z248	Overseas	Argentina	C1	G1	G2	var. fastigiata
Z249	Overseas	Israel	C3	G2	G3	var. hypogaea
Z250	Overseas	Mexico	C3	G2	G3	var. hypogaea
Z251	Overseas	Argentina	C1	G1	G2	var. fastigiata
Z254	Overseas	Israel	C3	G2	G3	var. hypogaea[1]
Z255	Overseas	Thailand	C1	G1	G2	var. fastigiata[2]
Z257	Overseas	China	C3	G2	G3	var. vulgaris
Z258	Overseas	ND	C1	G1	G2	var. fastigiata[1]
Z259	Overseas	Bolivia	C1	G1	G2	var. fastigiata[2]

Z260	Overseas	Nigeria	C3	G2	G3	var. hypogaea
Z261	Overseas	India	C2	G1	G1	var. vulgaris
Z262	Overseas	Zimbabwe	C2	G1	G1	var. vulgaris[2]
Z263	Overseas	Zimbabwe	C1	G1	G1	var. fastigiata
Z264	Overseas	Paraguay	C1	G1	G2	var. fastigiata[1]
Z265	Overseas	ND	C1	G1	G2	var. fastigiata[1]
Z266	Overseas	Paraguay	C1	G1	G1	var. fastigiata
Z267	Overseas	Brazil	C2	G1	G1	var. vulgaris[2]
Z268	Overseas	Burkina Faso	C3	G2	G3	var. hypogaea
Z269	Overseas	Bolivia	C3	G2	G3	var. hypogaea[1]
Z270	Overseas	Bolivia	C3	G2	G3	var. hypogaea[1]
Z272	Overseas	Colombia	C2	G1	G1	var. vulgaris
Z273	Landrace	China	C3	G2	G3	var. hypogaea
Z275	Landrace	China	C3	G2	G3	var. hypogaea
Z280	Landrace	China	C3	G2	G3	var. hypogaea
Z281	Landrace	China	C3	G2	G3	var. hirsuta
Z282	Landrace	China	C3	G2	G3	var. hypogaea
Z283	Landrace	China	C3	G2	G3	var. hypogaea
Z284	Landrace	China	C1	G1	G1	var. fastigiata
Z285	Landrace	China	C3	G2	G3	var. hypogaea[1]
Z287	Landrace	China	C3	G2	G3	var. hypogaea[1]
Z288	Landrace	China	C3	G2	G3	var. hypogaea[1]
Z291	Landrace	China	C3	G2	G3	var. hypogaea[1]
Z292	Landrace	China	C3	G2	G3	var. hirsuta[2]
Z294	Landrace	China	C3	G2	G3	var. hypogaea
Z299	Landrace	China	C3	G2	G3	var. hypogaea
Z300	Landrace	China	C3	G2	G3	var. hypogaea

Z306	Landrace	China	C3	G2	G3	var. hypogaea
Z309	Landrace	China	C3	G2	G3	var. hypogaea
Z319	Landrace	China	C3	G2	G3	var. hypogaea
Z323	Landrace	China	C3	G2	G3	var. hypogaea[1]
Z324	Landrace	China	C3	G2	G3	var. hirsuta
Z326	Landrace	China	C3	G2	G3	var. hypogaea
Z329	Landrace	China	C3	G2	G3	var. hypogaea[1]
Z330	Landrace	China	C2	G1	G1	var. vulgaris[1]
Z331	Landrace	China	C3	G2	G3	var. hypogaea[1]
Z332	Landrace	China	C2	G1	G1	var. vulgaris[1]
Z333	Landrace	China	C3	G2	G3	var. hirsuta
Z338	Landrace	China	C3	G2	G3	var. hypogaea
Z345	Landrace	China	C3	G2	G3	var. hypogaea
Z346	Landrace	China	C3	G2	G3	var. hirsuta
Z350	Landrace	China	C3	G2	G3	var. hypogaea
Z358	Landrace	China	C3	G2	G3	var. hypogaea
Z373	Landrace	China	C3	G2	G3	var. hirsuta
Z376	Landrace	China	C2	G1	G1	var. hypogaea
Z377	Breeding Line	China	C3	G1	G1	var. vulgaris
Z379	Breeding Line	China	C3	G2	G3	var. hypogaea[1]
Z382	Breeding Line	China	C3	G2	G3	var. hypogaea
Z383	Breeding Line	China	C2	G2	G3	var. vulgaris
Z384	Breeding Line	China	C3	G2	G3	irregular type
Z385	Breeding Line	China	C3	G2	G3	var. hypogaea
Z388	Breeding Line	China	C3	G2	G3	var. hypogaea
Z389	Breeding Line	China	C3	G2	G3	var. vulgaris
Z390	Breeding Line	China	C3	G2	G3	var. hypogaea
Z391	Breeding Line	China	C3	G2	G3	var. hypogaea

Z396	Breeding Line	China	C3	G2	G3	var. hypogaea[1]
Z399	Breeding Line	China	C3	G2	G3	var. hypogaea
Z402	Breeding Line	China	C3	G2	G3	var. hypogaea[1]
Z404	Breeding Line	China	C3	G1	G1	var. vulgaris
Z405	Breeding Line	China	C3	G2	G3	var. hypogaea
Z406	Breeding Line	China	C3	G2	G3	var. hypogaea
Z409	Breeding Line	China	C3	G2	G3	var. hypogaea
Z411	Breeding Line	China	C3	G2	G3	var. hypogaea[1]
Z421	Breeding Line	China	C3	G2	G3	var. hypogaea
Z422	Breeding Line	China	C3	G2	G3	var. hypogaea
Z423	Breeding Line	China	C3	G2	G3	var. hypogaea[1]
Z424	Breeding Line	China	C3	G2	G3	var. hypogaea
Z425	Breeding Line	China	C3	G2	G3	var. vulgaris
Z433	Breeding Line	China	C3	G2	G3	var. hypogaea
Z435	Breeding Line	China	C3	G2	G3	var. hypogaea
Z436	Breeding Line	China	C3	G2	G3	var. hypogaea
Z440	Breeding Line	China	C3	G2	G3	var. vulgaris
Z445	Breeding Line	China	C3	G2	G3	var. vulgaris
Z446	Breeding Line	China	C3	G2	G3	var. hypogaea
Z448	Breeding Line	China	C3	G2	G3	var. vulgaris
Z680	Breeding Line	China	C3	G2	G3	irregular type
Z681	Breeding Line	China	C3	G2	G3	var. hypogaea[1]
Z685	Breeding Line	China	C3	G2	G3	var. hypogaea[1]
Z687	Breeding Line	China	C3	G2	G3	irregular type
Z689	Breeding Line	China	C2	G1	G1	var. vulgaris
Z693	Breeding Line	China	C3	G2	G3	irregular type
Z695	Breeding Line	China	C3	G2	G3	var. hypogaea
Z696	Breeding Line	China	C3	G2	G3	var. hypogaea

Z839	Overseas	Japan	C3	G2	G3	var. hypogaea
Z840	Landrace	China	C2	G1	G1	var. vulgaris
Z847	Breeding Line	China	C3	G1	G1	var. vulgaris
Z851	Breeding Line	China	C3	G2	G3	var. hypogaea
Z871	Breeding Line	China	C3	G2	G3	var. hypogaea
Z872	Landrace	China	C3	G2	G3	var. hirsuta[1]
Z873	Breeding Line	China	C3	G2	G3	var. vulgaris
Z884	Breeding Line	China	C3	G2	G3	var. hypogaea
Z885	Breeding Line	China	C2	G1	G1	var. vulgaris
Z899	Breeding Line	China	C3	G2	G3	var. hypogaea
Z901	Landrace	China	C3	G2	G3	var. hirsuta
Z904	Landrace	China	C3	G2	G3	var. hypogaea
Z905	Overseas	Pakistan	C3	G2	G3	var. hirsuta[1]
Z906	Overseas	ND	C1	G1	G2	var. fastigiata[1]
Z907	Breeding Line	China	C3	G2	G3	var. hypogaea
Z908	Landrace	China	C3	G2	G3	var. vulgaris
Z909	Overseas	USA	C3	G2	G3	var. hirsuta[3]
Z912	Landrace	China	C2	G1	G1	var. vulgaris[1]
Z915	Landrace	China	C3	G2	G3	var. hirsuta
Z916	Landrace	China	C3	G2	G3	var. hypogaea[1]
Z921	Landrace	China	C2	G1	G1	var. vulgaris

[1] Not defined; [2] Within subspecies; [3] Between subspecies
